# Spatial biases during mental arithmetic: evidence from eye movements on a blank screen

**DOI:** 10.3389/fpsyg.2015.00012

**Published:** 2015-01-22

**Authors:** Matthias Hartmann, Fred W. Mast, Martin H. Fischer

**Affiliations:** ^1^Division of Cognitive Sciences, University of PotsdamPotsdam, Germany; ^2^Department of Psychology, University of BernBern, Switzerland

**Keywords:** mental arithmetic, eye movements, mental number line, operational momentum, embodied cognition, grounded cognition

## Abstract

While the influence of spatial-numerical associations in number categorization tasks has been well established, their role in mental arithmetic is less clear. It has been hypothesized that mental addition leads to rightward and upward shifts of spatial attention (along the “mental number line”), whereas subtraction leads to leftward and downward shifts. We addressed this hypothesis by analyzing spontaneous eye movements during mental arithmetic. Participants solved verbally presented arithmetic problems (e.g., 2 + 7, 8–3) aloud while looking at a blank screen. We found that eye movements reflected spatial biases in the ongoing mental operation: Gaze position shifted more upward when participants solved addition compared to subtraction problems, and the horizontal gaze position was partly determined by the magnitude of the operands. Interestingly, the difference between addition and subtraction trials was driven by the operator (plus vs. minus) but was not influenced by the computational process. Thus, our results do not support the idea of a mental movement toward the solution during arithmetic but indicate a semantic association between operation and space.

In Western cultures small numbers are typically represented to the left of larger numbers, both in external space (e.g., on rulers and timetables) and in cognitive space, following the concept of the “mental number line” (e.g., Dehaene et al., 1993; Hubbard et al., 2005; Fischer and Shaki, 2014a). The pervasive small-left and large-right-association is captured by the SNARC (spatial-numerical association of response codes) effect, showing left-sided response facilitation for small numbers and right-sided response facilitation for large numbers. Spatial-numerical associations have been well established in the horizontal dimension of space (see Fischer and Shaki, 2014a, for a review), and have recently been extended to vertical space (e.g., Ito and Hatta, 2004; Loetscher et al., 2010; Grade et al., 2012; Hartmann et al., 2012, 2014a; Holmes and Lourenco, 2012; Shaki and Fischer, 2012; Fischer, 2012; Winter and Matlock, 2013). For example, when participants name numbers at random, they generate smaller numbers during downward when compared to upward body motion (Hartmann et al., 2012; Winter and Matlock, 2013). These spatial-numerical associations are in line with the embodied approach of knowledge representation, according to which our sensory and motor experiences during concept acquisition remain associated with these concepts (e.g., Barsalou, 2008; Pulvermüller, 2013). In the case of numbers, their horizontal association has been attributed to reading and writing, as well as finger counting habits, while the vertical association might reflect the experience that “more” usually corresponds to higher space (e.g., Lakoff and Johnson, 1980; Zebian, 2005; Fischer and Brugger, 2011; Göbel et al., 2011; Fischer, 2012; Holmes and Lourenco, 2012; but see Hartmann et al., 2014a).

Spatial biases during number processing have predominantly been studied by means of simple number categorization tasks (small vs. large, even vs. odd). The role of spatial biases during more complex numerical tasks, such as mental arithmetic, is less clear (Fischer and Shaki, 2014b). In a seminal study, McCrink et al. (2007) asked participants to judge whether a final set of objects was the correct result of a preceding addition or subtraction process. Participants were more likely to accept a solution with too many objects for addition and with too few objects for subtraction. This systematic bias has been labeled “operational momentum effect.” In the perceptual domain, the “representational momentum effect” describes the misperception of the vanishing position of a moving dot. Particularly, the vanishing position is perceived as being further along the dot's movement trajectory. In analogy, the operational momentum effect suggests that addition is conceptualized as excessive rightward movement and subtraction as excessive leftward movement along the mental number line (McCrink et al., 2007; Knops et al., 2009b). Further empirical evidence for such a spatial process during mental arithmetic comes from Wiemers et al. (2014) who found that addition and subtraction problems were solved faster when participants made arm movements congruent with the hypothesized movements along the mental number line (i.e., rightward or upward for addition, and leftward or downward for subtraction). Similarly, adding was found to be easier when participants rode upward in an elevator whereas riding downward facilitated subtracting (Lugli et al., 2013). Moreover, Marghetis et al. (2014) observed systematic leftward and rightward deflections in participants' hand trajectories when they indicated the results of addition and subtraction problems with a mouse cursor movement. Furthermore, Masson and Pesenti (2014) found that targets in the left visual field were detected faster after solving subtraction problems whereas targets in the right visual field were detected faster after solving addition problems. Finally, patients suffering from hemispatial neglect after right-hemispheric brain lesion show selective deficits for subtraction but not for addition problems, in line with their selective deficit in orienting attention toward the left side of space (Dormal et al., 2014).

Despite this empirical evidence, the exact mechanism leading to the spatial bias during mental arithmetic is far from clear (Fischer and Shaki, 2014b). First of all, the idea of moving leftward (for subtraction) and rightward (for addition) along the mental number line is only one of several possible explanations for the operational momentum effect (for a discussion of alternative accounts see Knops et al., 2013, 2014; Marghetis et al., 2014; Fischer and Shaki, 2014a). Moreover, most evidence for a spatial bias in mental arithmetic comes from tasks that imposed a specific spatial setting, for example by requiring participants to respond with a left or a right key (i.e., Masson and Pesenti, 2014), or involving movements along a specific spatial axis (Pinhas and Fischer, 2008; Lugli et al., 2013; Marghetis et al., 2014; Wiemers et al., 2014). These bipolar spatial assignments imposed by the task setting might also shape the spatial bias during mental arithmetic (Proctor and Cho, 2006). Lastly, Pinhas et al. (2014) showed that the operation sign itself (±) has a spatial connotation (plus-right and minus-left) in a speeded manual classification task. Therefore, it is unclear to what extent the reported results reflect spatial biases induced by the actual mental computation (i.e., the activated magnitudes) or rather by the semantic spatial association of the operation sign.

The aim of this study is to further investigate spatial biases during mental arithmetic by means of eye movements. Eye movements reflect the spatial focus of attention (e.g., Sheliga et al., 1994; Corbetta et al., 1998) and have been used to study the spatial character of ongoing mental processes with high temporal resolution (e.g., Spivey and Geng, 2001; Grant and Spivey, 2003; Altmann, 2004; Van Gompel et al., 2007; Huette et al., 2014; Johansson and Johansson, 2014; Hartmann et al., 2014b). Eye movement studies have contributed to the understanding of cognitive processes involved in numerical tasks (Suppes, 1990; Loetscher and Brugger, 2007; Loetscher et al., 2008; Moeller et al., 2011; Sullivan et al., 2011; Schneider et al., 2012; Zhou et al., 2012; Chesney et al., 2013; Van Viersen et al., 2013; Klein et al., 2014; Huber et al., 2014a,b). Most importantly in the context of the present study, spontaneous eye movements (i.e., eye movements that are not triggered in response to a perceptual event) follow spatial-numerical associations: Loetscher et al. (2010) were able to predict the magnitude of numbers in their participants' mind during random number generation, based on the direction and magnitude of spontaneous saccades occurring before the number was spoken out. Particularly, rightward and upward saccades were more frequent when the next number was larger than the previous one (see also Loetscher et al., 2008).

In this study, we analyzed spontaneous eye movements on a blank screen while participants solved verbally presented arithmetic problems. Based on horizontal and vertical spatial-numerical associations and on previous arithmetic-space compatibility effects (e.g., Lugli et al., 2013; Wiemers et al., 2014), we hypothesized that participants' gaze would shift rightward and upward during addition, and leftward and downward during subtraction. Crucially, analysis of the time course of the spatial bias will help to clarify the temporal dynamics of the spatial bias induced by the different elements involved in the operation (magnitude of the first and second operand, the operator, and the size of the solution). Moreover, our paradigm should help to further describe the nature of the spatial bias in mental arithmetic since no predefined spatial dimension was imposed by the stimulus or response arrangement.

## Method

### Participants

Twenty-five undergraduate students from the University of Bern participated in this study for course credit (19 women, mean age: 23.0, range: 19–45 years, three left-handed). Participants gave written informed consent prior to the study, and the study was approved by the local Ethics Committee. All participants had normal or corrected-to-normal visual acuity.

### Stimuli and procedure

The following operands were used: 2, 3, 4, 5, 6, 7, 8, 9. Eighteen pairs of different operands were selected for constructing the arithmetic problems (see Appendix). For both addition and subtraction trials, the 18 pairs were presented once in the original order and once in the reverse order, resulting in a total number of 72 unique problems. For the purpose of this study it was important that the magnitude of the first operand does not allow participants to predict which operation (addition vs. subtraction) will follow. In most previous studies, addition trials were more likely when the first operand was a small number, and subtraction more likely when the first operand was a large number (e.g., Pinhas and Fischer, 2008; Wiemers et al., 2014), which could induce predictive eye movements along the mental number line before the onset of the operator. We minimized this effect by choosing a similar amount of small and large numbers as first operand for addition and subtraction trials. As a result of this, half of the subtraction trials had a negative solution size. Negative numbers, when intermixed with positive numbers, are located on the left side of the mental number line (Fischer, 2003; Ganor-Stern et al., 2010).

Participants were seated 70 cm in front of the screen and instructed to solve as fast and accurately as possible an auditorily presented addition or subtraction problem. Auditory stimuli were presented via loudspeakers positioned 30 cm to the left and right side of the screen. At the beginning of each trial, a central fixation cross was presented for 1 s. The fixation cross was implemented to shift spatial attention to the center of the screen at the beginning of each trial. This allows to compare the development of spatial biases with respect to the center of the screen across trials.

The cross disappeared at the onset of the first operand and remained blank. Each audio file (first operand, operator, second operand) lasted 500 ms. The operator followed 750 ms after the offset of the first operand, and the second operand followed 750 ms after the offset of the operator. The time course of a trial is shown in **Figure 2**. Participants pressed the space bar as soon as they had solved each problem and at the same time speak out the solution. The solution was noted by the experimenter. The inter-stimulus interval (i.e., the time between offset of the second operand and onset of the fixation cross preceding the next trial) was 5 s.

### Apparatus

Eye movements were recorded with an SMI RED tracking system (SensoMotoric Instruments, Teltow, Germany). Eye gaze was registered with a sampling rate of 50 Hz, a spatial resolution of 0.1° and a gaze position accuracy of 0.5°. The stimuli were presented on a 17-inch screen (1280 × 1024 pixels) using Experiment Center Software and eye data were recorded with I-View X Software, both developed by SensoMotoric Instruments (SensoMotoric Instruments, Teltow, Germany). The primary output events of the eye tracker were fixations (the sample frequency of 50 Hz did not allow us to detect and analyze saccade latencies accurately). Fixations were extracted using Be-Gaze software (SensoMotoric Instruments, Teltow, Germany) and were defined by a minimum duration of 80 ms (4 samples) and a maximal dispersion of 100 pixels^1^.

### Statistical analysis

We first documented arithmetic performance of our participants to show that they complied with our instructions. Response times (RTs) were measured from the onset of the second operand. Trials with RTs larger than 3 s were excluded from further analysis (0.4%). For the eye movement data analyses, we defined the following three time windows: Time window 1 started at the onset of the first operand and ended with the onset of the operator (0—1250 ms). Time window 2 started with the onset of the operator and ended with the onset of the second operand (1250—2500 ms). Finally, Time window 3 started with the onset of the second operand and ended when participants pressed the response key (2500 ms—response time). Within each time window, we analyzed the position of the first fixation that participants initiated (i.e., after the onset of the first operand, the onset of the operator, and the onset of the second operand). Moreover, we also analyzed within the same time windows the horizontal and vertical position at each sample of the full data stream (allowing to describe spatial biases with a high temporal resolution). One participant was excluded from the analysis of eye movements due to data loss. Trials where the initial fixation position was not on the central fixation position (more than ± 1° of visual angle) were excluded from the analysis (4% of trials).

#### Analysis of the first fixation

In order to recognize the content of the audio file (numbers and operator) it was necessary to hear approximately the first 150 ms of the audio file. We therefore excluded fixations that were initiated within the first 150 ms from the onset of the audio file. Moreover, fixations outside of the screen were excluded from this analysis (4.9% of fixations). Importantly, the position of the first fixation was always expressed relative to the x and y coordinates of the sample at the onset of the respective time window; this normalization controls for differences in the previous trial history and allows the comparison of fixation positions across trials.

For each time window, a repeated measures regression (using a linear mixed model approach with random intercepts for participants and fixed effects for the predictors) was computed for the horizontal and vertical position of the first fixation. For Time window 1, the magnitude of the first operand was used as predictor. For Time window 2, again the magnitude of the first operand was used as predictor (since this factor could still influence behavior in later time windows) along with the operator (+, −). For Time window 3, the magnitude of the first operand, the operator, the magnitude of the second operand, and the solution size were predictors. We also included the interaction between the operator and the solution size as predictor. This interaction captures a possible rightward shift for larger solution sizes during addition trials and a possible leftward shift for smaller (and negative) solution sizes during subtraction trials. For all analyses, the variables magnitude of operands and solution size were treated as covariates since we were interested in the *linear* effect of number magnitude on gaze position.

#### Analysis of the full gaze stream

In order to get a more fine-grained picture of the spatial biases induced by the different elements (operands, operator, solution size), we analyzed the horizontal and vertical gaze position for each sample of the raw data gaze stream (i.e., every 20 ms). Gaze positions recorded during eye blinks, as well as the samples immediately before and after a blink, were treated as missing values. All missing values, including samples with coordinates outside of the screen or signal loss, were replaced by linear interpolation. Trials that consisted of more than 30% interpolated data were then removed from the analysis (3.3%). In order to average and compare gaze position in Time window 3 across trials with different numbers of recorded samples (depending on the response time), data in Time window 3 were time-normalized into 60 samples (60 samples equals 1200 ms which roughly corresponds to the mean RT found in our sample) using linear interpolation. The same analyses as described above for the first fixations were then performed on each sample. The analyses for Time windows 2 and 3 included all samples from the beginning of the time window until the end of the trial, corrected for the position at the beginning of the respective time window. For the analyses performed on the samples of the gaze stream, we only considered effects as statistically significant when the *p*-values of at least 10 consecutive samples were below 0.05 (corresponding to a 200 ms interval; see Mathot et al., 2013 for a similar approach).

## Results

### Arithmetic performance

**Figure 1:**
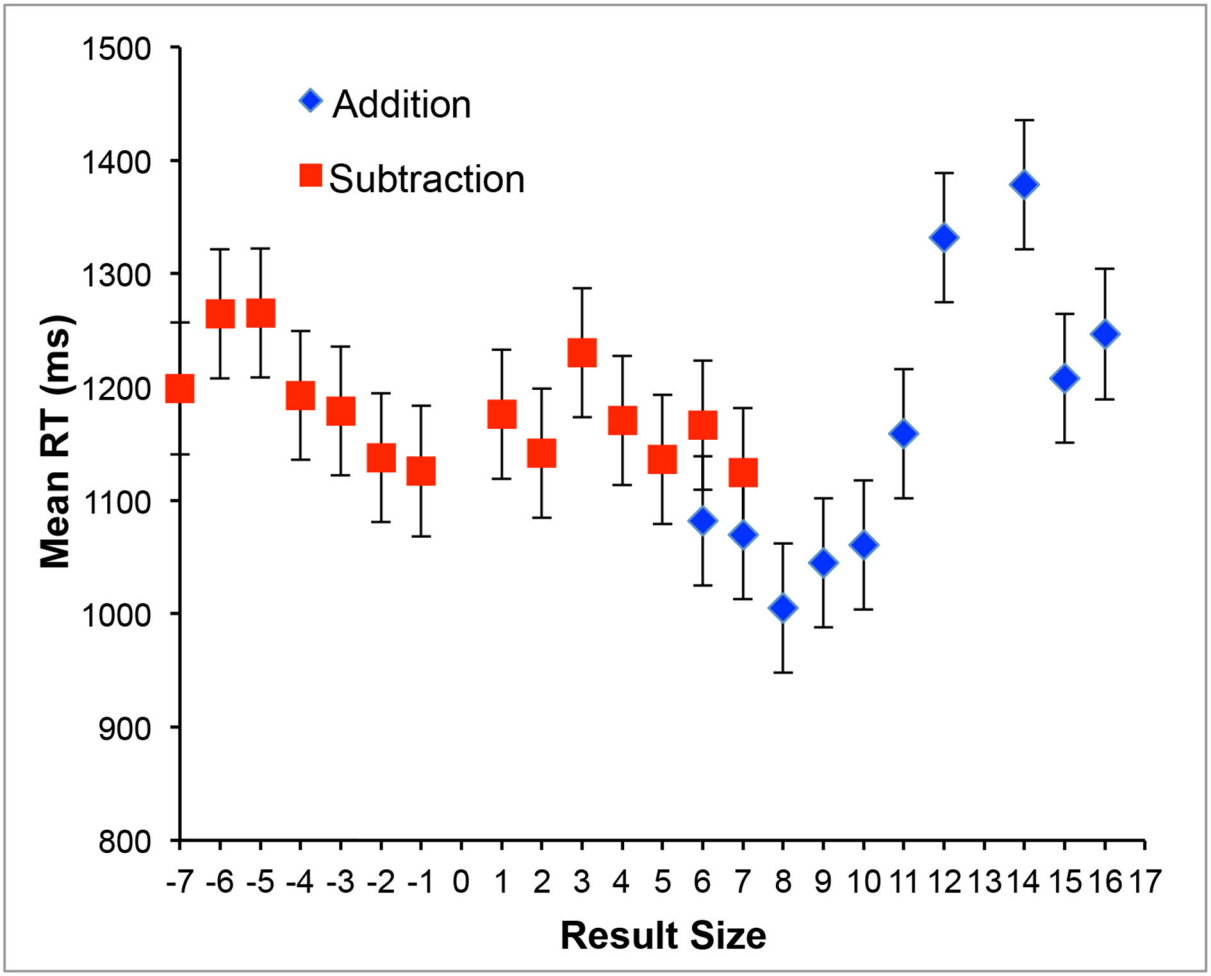
**Mean Response Times (RT) for the different result sizes for addition and subtraction trials**. Error bars depict ± 1 *SEM*.

Error rate was low (1.1%) and was not further analyzed. Mean RTs were on average higher for subtraction than for addition trials (1178 vs. 1148 ms)^2^, as revealed by a paired *t*-test, *t*_(24)_ = 2.51, *p* = 0.019. RTs for the different result sizes for addition and subtraction trials are illustrated in Figure 1. The findings that RTs were generally higher for subtraction than for addition trials and that RTs increased with increasing (absolute) result sizes (see Figure 1) are both in line with general findings about mental arithmetic (Ashcraft, 1992; Campbell, 2005). Thus, our participants complied with the task instruction to solve mental arithmetic problems.

### Gaze position

A full statistical report for the analysis of the first fixation in each time window is presented in Table 1. Here we only report the most important findings.

**Table 1:** **Statistical report of the linear mixed model analyses on the first fixation position after the onset of the operands and operator**.

**Predictor**	**Horizontal fixation position**			**Vertical fixation position**		
	**Estimation**	***F***	***p***	**Estimation**	***F***	***p***
**TIME WINDOW 1**						
Operand 1	−1.0 (1.1)	0.84	0.360	−1.7 (1.0)	2.76	0.097
**TIME WINDOW 2**						
Operand 1	−1.1 (1.3)	0.74	0.389	−0.1 (1.1)	0.01	0.954
Operator	4.6 (6.0)	0.60	0.439	11.5 (5.1)	5.01^*^	0.025
**TIME WINDOW 3**						
Operand 1	0.2 (2.0)	0.01	0.911	−0.7 (1.9)	0.14	0.709
Operator	−1.5.8 (25.0)	0.01	0.953	−8.4 (23.1)	0.13	0.715
Operand 2	−1.5 (2.0)	0.59	0.442	−1.0 (1.9)	0.28	0.595
Solution size (SS)	−2.2 (1.5)	0.23	0.629	1.5 (1.4)	0.27	0.605
SS × Operator	3.2 (2.7)	1.36	0.244	−1.7 (2.5)	0.44	0.506

* p < 0.05. The unit of the estimates is pixel; Operand 1, Operand 2, and solution size are treated as covariates.

#### Time window 1

Participants initiated in 71% of trials a new eye fixation in Time window 1. There was no linear influence of the magnitude of the first operand on the position of the first fixation (horizontal gaze position: *p* = 0.360; vertical gaze position: *p* = 0.097). The analysis of the full gaze stream confirmed that there was no significant effect of the magnitude of the first operand.

#### Time window 2

Participants initiated in 74.4% of trials a new eye fixation in Time window 2. The operator was a significant predictor for the vertical gaze position, *F*_(1, 1166)_ = 5.01, *p* = 0.025, but not for the horizontal gaze position, *F*_(1, 1166)_ = 0.60, *p* = 0.439: Gaze position of the first fixation initiated after the onset of the operator was located 12 pixels more upward for “plus” when compared to “minus.”

**Figure 2:**
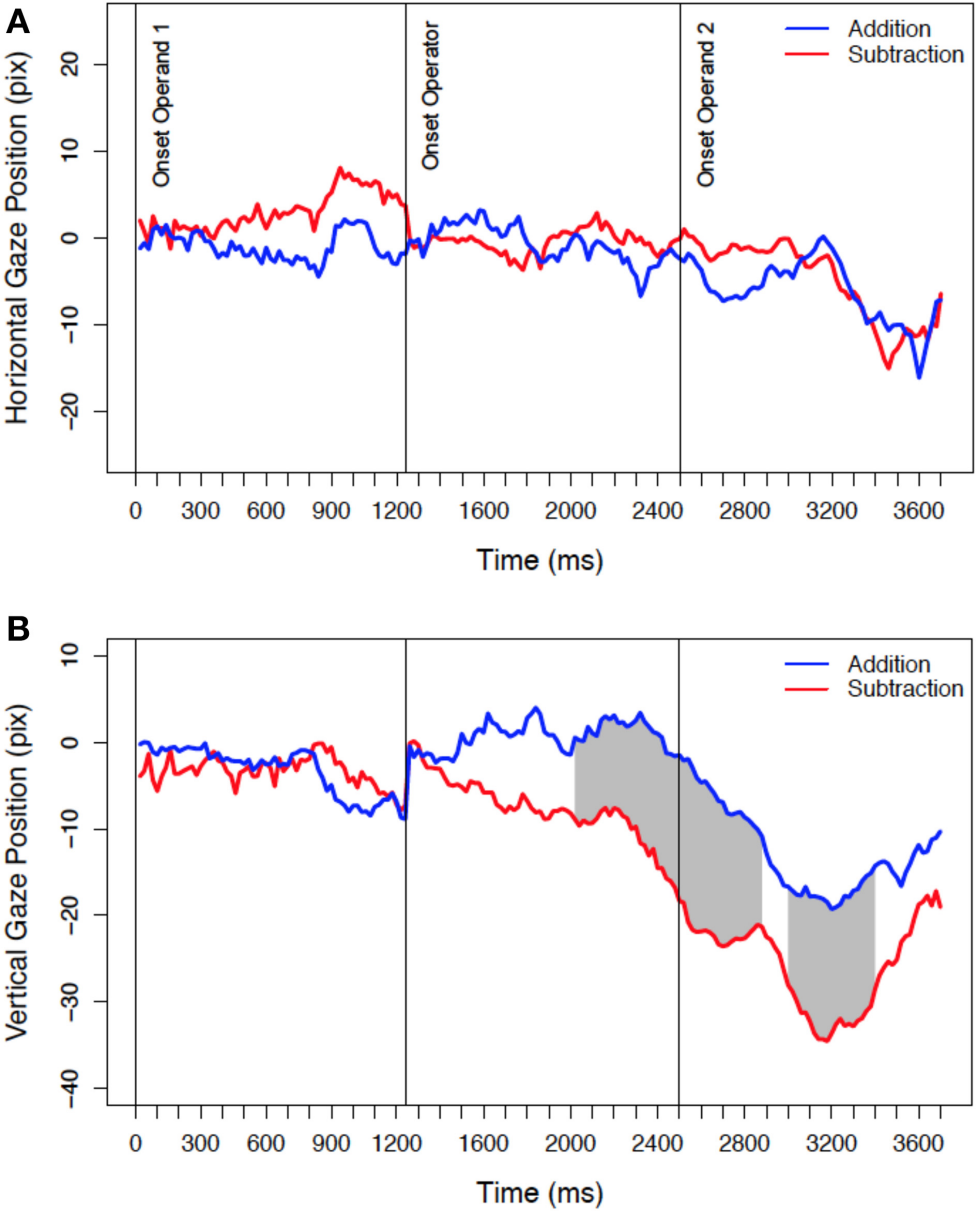
**Mean horizontal (A) and vertical (B) gaze position during mental arithmetic**. Zero at the y-axis represents the center of the screen, and negative values left screen **(A)** or lower screen **(B)** positions. Data is corrected for the position at the onset of the operator and shows the development of the difference between addition and subtraction trials until the response is given (last data point). The gray area indicates statistically significant differences (significance criterion: *p* < 0.05 for at least 10 consecutive samples).

The analysis of the full gaze stream confirmed the spatial bias induced by the operator for the vertical gaze position, as well as the absence of a bias for the horizontal gaze position (see Figure 2). Differences between addition and subtraction trials for the vertical gaze position start to develop shortly after the onset of the operator and remain for a large part of the trial. Significant differences between addition and subtraction trials are represented by the gray areas in Figure 2B. The first significant difference was detected 760 ms after the onset of the operator.

#### Time window 3

Participants initiated in 75.3% of trials a new eye fixation in Time window 3. Remarkably, none of the variables (magnitude of the first operand, operator, magnitude of the second operand, solution size, and the interaction between solution size and operator) predicted the position of the first fixation after the onset of the second operand.

**Figure 3:**
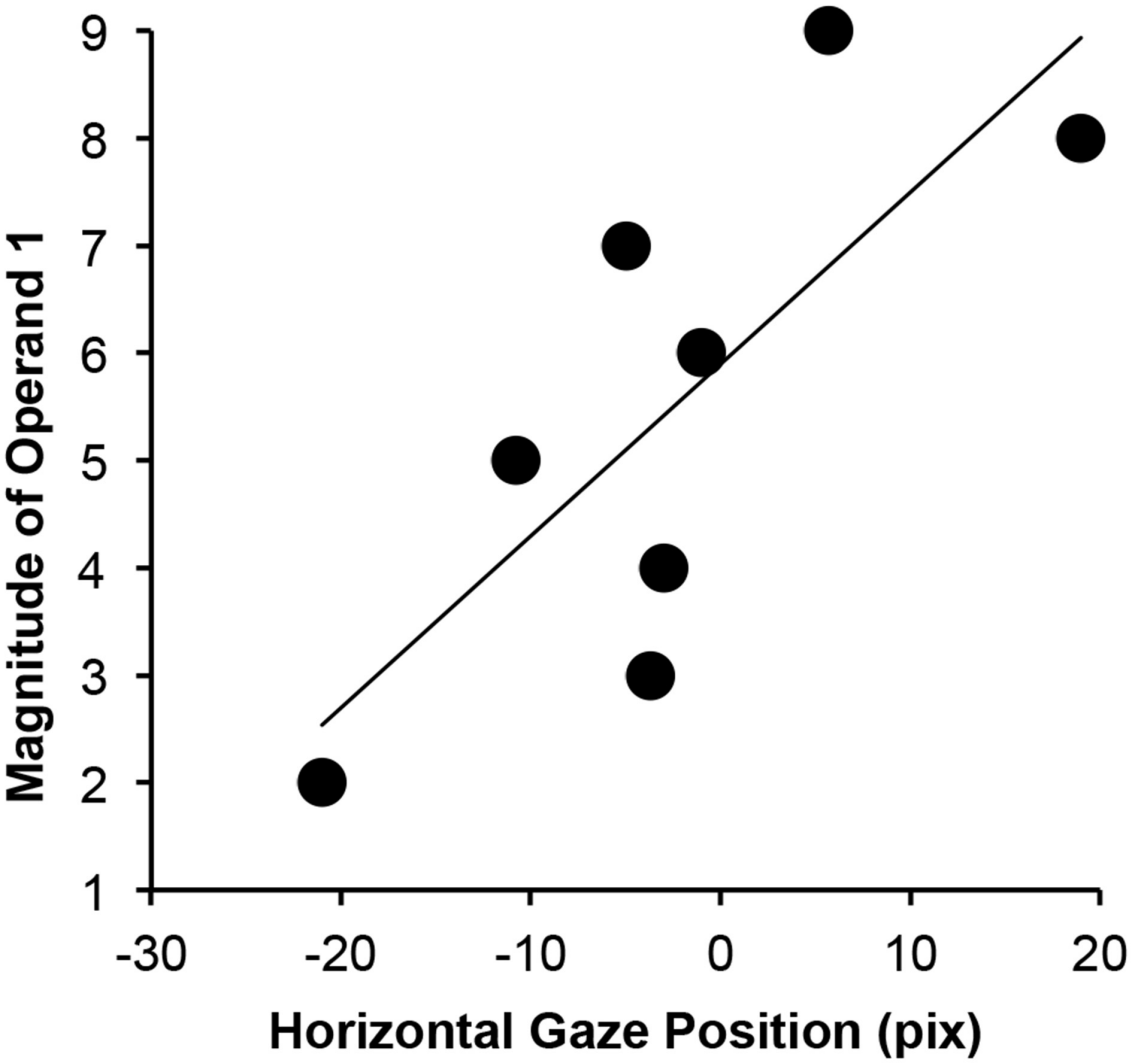
**Horizontal gaze position as a function of the magnitude of the first operand**.

The analysis of the continuous gaze stream (including the last sample where participants gave their responses) revealed that there was no effect of the operator, the magnitude of the second operand, the solution, and the interaction between the solution and the operator. Importantly, the effect of the operator found in Time window 2 is no longer significant when positions are corrected for differences at the onset of Time window 3 (as it was done for this analysis). Thus, gaze position was not systematically influenced by the computational process. There was, however, a trend for an effect of the magnitude of the first operand (a series of six consecutive samples with *ps* < 0.052) on the horizontal gaze position. This time period was identified between 2940 and 3080 ms after the onset of the first operand, or, respectively, between 440 and 580 ms after the onset of the second operand (corresponding to 25–33% of the progress for the time-normalized computation process). When we repeated the linear mixed effect model analysis for this specific time interval, the estimated linear effect was 3.4 (*SE* = 0.6; *p* < 0.001), clearly indicating that gaze position was shifted more rightward as a function of number magnitude, as shown in Figure 3.

## Discussion

In this study we analyzed spontaneous eye movements on a blank screen when participants solved addition and subtraction problems. We found that the gaze was directed more upward during addition than during subtraction trials, and horizontal gaze position was partly determined by the magnitude of the operand. The former finding is in line with the small-down and large-up orientation of the vertical mental number line (e.g., Grade et al., 2012; Hartmann et al., 2014a; Experiment 1; Loetscher et al., 2010; Hartmann et al., 2012; Holmes and Lourenco, 2012; Winter and Matlock, 2013) and supports the view that addition is associated with upper space and subtraction with lower space (Lugli et al., 2013; Wiemers et al., 2014). The latter finding, a more rightward gaze position for larger magnitudes of the operand, is in line with the small-left and large-right orientation of the horizontal mental number line (e.g., Dehaene et al., 1993; Fischer and Shaki, 2014a), and confirms that numbers can induce shifts of spatial attention (Fischer et al., 2003). Thus, our results show that spontaneous eye movements reflect systematic spatial biases during mental arithmetic and provide new evidence for an active role of eye movements for magnitude processing. Of particular relevance in the context of numerical cognition are the findings that the difference between addition and subtraction trials was induced by the operator, and that this effect manifested itself in the vertical but not in the horizontal dimension of space. These two aspects are now discussed in more detail.

Previous findings of operational momentum effects during mental arithmetic (e.g., McCrink et al., 2007; Pinhas and Fischer, 2008; Knops et al., 2009b) and motion-arithmetic compatibility effects (Lugli et al., 2013; Marghetis et al., 2014; Wiemers et al., 2014) suggest that mental addition and subtraction is accompanied by a mental movement along the number line. Do our results support this idea? In this study, the difference between addition and subtraction trials developed shortly after the onset of the operator, before the second operand was presented and consequently before the computational process was initiated. The difference between addition and subtraction trials remained present from there on. Importantly, when gaze position was controlled for the differences at the onset of the second operand (see analysis of Time window 3), there was no further contribution from the operator to the spatial bias in that time window. This clearly shows that the difference between addition and subtraction trials can only be attributed to the operator (i.e., the onset of the operator in Time window 2) and not to the addition or subtraction process *per se* (i.e., the computational process that took place in Time window 3). A spatial bias induced by the computational process would also result in an interaction between the operator and the solution size, which was absent in all our analyses. We therefore conclude that the addition-up and subtraction-down association we found reflects a semantic operation (addition vs. subtraction) spatial association effect rather than the consequence of a spatial shift that occurred *during* computation. Thus, our results do not support the idea that adding magnitudes involves a simulated rightward or upward movement, and subtracting a leftward or downward movement along the mental number line, at least not when addition and subtraction problems are solved on a trial-by-trial basis (note that mental movements or OM effects might be more pronounced for continuous counting). Instead, our results confirm an operation sign spatial association (OSSA) effect that was recently demonstrated by Pinhas et al. (2014) with manual responses.

Our replication and extension of Pinhas et al.'s results has important implications. First of all, we found an OSSA effect for auditorily presented operators. This shows that the perception of the operation *sign* is not mandatory, and suggest that the semantic processing of the operator is the crucial aspect. Consequently, the OSSA effect should be renamed into OSA (operator spatial association) effect. Moreover, our results suggest that the principal role of space during mental arithmetic might be the activation of metaphorical magnitude concepts, such as “more is up” (e.g., Lakoff and Johnson, 1980; Fischer, 2012; Holmes and Lourenco, 2012). The activation of such a spatial concept might support the task by providing an intuitive spatial reference for the solution, or in other words, by providing a “rough sense of expected magnitude against which the algorithmically derived solution can be compared” (Marghetis et al., 2014, p. 13; see also Stevenson and Carlson, 2003). Thus, it is conceivable that participants changed their vertical gaze position depending on the operator because the result of the computation will be smaller (down) or larger (up) than the current reference (i.e., the magnitude of the first operand). Marghetis et al. (2014) also found that the operator induced the strongest spatial bias in hand trajectories performed during mental arithmetic (see Figure 4 in Marghetis et al., 2014). To put it in a nutshell, there is a systematic spatial bias during mental arithmetic, but this bias might not primarily be constituted by simulating an exact movement along the mental number line but rather by the spatialization of an approximate sense of quantity, which might already be triggered by the operator.

Another interesting aspect of our findings is that the difference between addition and subtraction was only present in the vertical dimension of space. Based on the fact that the mental number line is running from left to right in ascending order (in Western cultures), we also expected an addition-right and subtraction-left association. Indeed, many studies point to such an association, for both non-symbolic (McCrink et al., 2007; Knops et al., 2009a,b) and symbolic (Pinhas and Fischer, 2008; Knops et al., 2009a; Marghetis et al., 2014; Masson and Pesenti, 2014; Pinhas et al., 2014; Wiemers et al., 2014) arithmetic. What are possible explanations for the absence of such an effect in our study? First of all, previous studies showing a spatial-arithmetic association in the horizontal dimension imposed an explicit horizontal component in their tasks. For example, target stimuli or response keys were arranged on a horizontal line on the screen or on the table, respectively (Pinhas and Fischer, 2008; Marghetis et al., 2014; Masson and Pesenti, 2014; Pinhas et al., 2014), or required participants to make hand movements along a horizontal line (Pinhas and Fischer, 2008; Wiemers et al., 2014). Thus, in all these studies, the horizontal dimension of space was made salient to participants, which might facilitate the use of the horizontal axis of space in participants' task representation. In the present study, we used a blank screen paradigm with no predefined spatial arrangements of stimulus or responses. We argue that, in cases where no predefined spatial frame of reference is provided, participants recruit those spatial associations that are grounded deepest in their cognitive system. The experience that “more” usually corresponds to upper space might constitute a fundamental concept of our cognitive system (e.g., Lakoff and Johnson, 1980; Zebian, 2005; Göbel et al., 2011; Fischer, 2012; Holmes and Lourenco, 2012; but see Hartmann et al., 2014a). For example, adding objects or water to a bowl raises the horizontal level. Moreover, larger objects occupy more upper space than smaller objects (e.g., skyscraper vs. cottage). Because such observations are universal and accompany the development of our cognitive system since early childhood, the concept of “more is up” might be more hard-wired (or grounded) than the concept of “more is right,” which shows a great deal of flexibility across cultures and task demands (e.g., Bächtold et al., 1998; Zebian, 2005; Ristic et al., 2006; Shaki et al., 2009; Fischer et al., 2010). For these reasons, it might be more intuitive for participants to use the upper and lower space and not the left and right space in order to conceptualize addition and subtraction during mental arithmetic. In line with this view, the spatial-arithmetic compatibility effects found in Wiemers et al.'s (2014) study were more pronounced for the vertical than for the horizontal spatial axis.

As a last point, we want to discuss the effect of the magnitude of the first operand for the horizontal gaze position. As this effect did not reach our significance criterion, we do not want to over-interpret this effect but nevertheless point to some interesting aspects. Interestingly, the spatial bias induced by the magnitude of the first operand did not obtain in the time window where the magnitude was perceived. Rather, the effect was only evident after the onset of the second operand. This suggest that perceiving numbers does not always automatically shift spatial attention along the mental number line (Fischer et al., 2003) but requires that the number is extensively processed (see Fischer and Knops, 2014; Zanolie and Pecher, 2014, for a discussion). In this case, it might reflect the re-activation of the number meaning of the first operand in order to initiate the computational process that was triggered by the additional information provided by the operator and the second operand. A similarly delayed influence of the magnitude of the first operand was also found by Marghetis et al. (2014).

### Outlook

A possible limitation of this study was that we used relatively simple arithmetic problems that can potentially be solved by memory retrieval (Ashcraft, 1992; Campbell, 2005). It is conceivable that more complex problems that rely more heavily on computation would recruit more pronounced spatial processing (and possibly to operational momentum effects). However, recent work from Fayol and Thevenot (2012) suggests that seemingly simple arithmetic problems (such as 3 + 4) can also activate a procedural strategy. Further studies are needed in order to draw final conclusions about the nature of spatial biases in mental arithmetic and its role for different levels of complexity of arithmetic problems and formats (symbolic vs. non-symbolic). The use of methods that allow for a continuous tracking of the arithmetic process, such as eye or hand movement studies will be most fruitful for future research (Fischer and Hartmann, 2014; Marghetis et al., 2014). Moreover, future tasks should be designed in a way that allows the researcher to disentangle whether the spatial bias was induced by the computational process or by the operator alone, for example by including trials that do only contain operation signs but no operands (see Pinhas et al., 2014).

## Conclusion

We showed that spontaneous eye movements reflect spatial biases during mental arithmetic and highlighted an important role of the operator for inducing these spatial biases. On a global level, our results suggest that eye movements might play an important role in cognition because they translate abstract concepts, such as number magnitudes and arithmetic, into concrete spatial relationships, possibly in order to facilitate the understanding and mental manipulation of these concepts. Our results add to a growing body of research showing that apparent abstract mental processes are accompanied by sensorimotor processes, reflecting the embodied nature of knowledge representation (Gallese and Lakoff, 2005; Fischer, 2012).

### Conflict of interest statement

The authors declare that the research was conducted in the absence of any commercial or financial relationships that could be construed as a potential conflict of interest.

## Footnotes

^1^The algorithm checks the dispersion of consecutive data points in a moving window by summing the differences between the points' maximum and minimum x and y values ([max(x) − min(x)] + [max(y)-min(y)]). If the sum is below 100 pixels, the window represents a fixation and expands until the sum exceeds 100 pixels. The final window is registered as fixation at the centroid of the window points with the given onset time and duration.

^2^ Noteworthy, the two highest means were found for the addition trials with the result sizes 12 and 14. These were the problems 7 + 5 / 5 + 7 (for 12), and 6 + 8 / 8 + 6; 9 + 5 / 5 + 9 (for 14). These problems are characterized by a carry operation, which is known to increase RT (e.g., Imbo et al., 2007). The problems with even higher result sizes (15, 16) do also require a carry operation but these problems include the operand 9 (6 + 9 / 9 + 6 for 16 and 7 + 9 / 9 + 7 for 17), which facilitates the carry operation (9 is close to 10 and allows for alternative strategies, such as “add 10 and subtract 1”).
