# Supplementary material for: Spatial biases during mental arithmetic: evidence from eye movements on a blank screen
Source: Front Psychol. 2015 Jan 22;6:12. doi: 10.3389/fpsyg.2015.00012 (PMC4302709; doi:10.3389/fpsyg.2015.00012)
Supplement: Supplementary file 1 [file DataSheet1.DOCX]

Appendix

Stimulus list

| Addition trials | Subtraction trials |
| --- | --- |
| 2 + 4 | 2 – 6 |
| 2 + 5 | 2 – 7 |
| 2 + 6 | 2 – 8 |
| 2 + 8 | 2 – 9 |
| 2 + 9 | 3 – 4 |
| 3 + 4 | 3 – 5 |
| 3 + 6 | 3 – 7 |
| 3 + 7 | 3 – 8 |
| 3 + 8 | 3 – 9 |
| 4 + 2 | 4 – 3 |
| 4 + 3 | 4 – 6 |
| 4 + 5 | 4 – 7 |
| 4 + 6 | 4 – 9 |
| 4 + 7 | 5 – 3 |
| 5 + 2 | 5 – 7 |
| 5 + 4 | 5 – 8 |
| 5 + 6 | 5 – 9 |
| 5 + 7 | 6 – 2 |
| 5 + 9 | 6 – 4 |
| 6 + 2 | 6 – 8 |
| 6 + 3 | 6 – 9 |
| 6 + 4 | 7 – 2 |
| 6 + 5 | 7 – 3 |
| 6 + 8 | 7 – 4 |
| 6 + 9 | 7 – 5 |
| 7 + 3 | 7 – 9 |
| 7 + 4 | 8 – 2 |
| 7 + 5 | 8 – 3 |
| 7 + 9 | 8 – 5 |
| 8 + 2 | 8 – 6 |
| 8 + 3 | 9 – 2 |
| 8 + 6 | 9 – 3 |
| 9 + 2 | 9 – 4 |
| 9 + 5 | 9 – 5 |
| 9 + 6 | 9 – 6 |
| 9 + 7 | 9 – 7 |
